# Who would students ask for help in academic cheating? Cross-sectional study of medical students in Croatia

**DOI:** 10.1186/s12909-014-0277-y

**Published:** 2014-12-30

**Authors:** Varja Đogaš, Ana Jerončić, Matko Marušić, Ana Marušić

**Affiliations:** Department of Psychological Medicine, University of Split, School of Medicine, Šoltanska 2 21000 Split, Croatia; Department of Research in Biomedicine and Health, University of Split, School of Medicine, Šoltanska 2, 21000 Split, Croatia

## Abstract

**Background:**

Academic cheating does not happen as an isolated action of an individual but is most often a collaborative practice. As there are few studies that looked at who are collaborators in cheating, we investigated medical students’ readiness to engage others in academic dishonest behaviours.

**Methods:**

In a cross-sectional survey study in Zagreb, Croatia, 592 medical students from the first, 3rd and 6th (final) study year anonymously answered a survey of readiness to ask family, friends, colleagues or strangers for help in 4 different forms of academic cheating or for 2 personal material favours. Stepwise multiple linear regression models (MLR) were used to evaluate potential factors influencing propensity for engaging others in these two types of behaviour.

**Results:**

Many students would ask another person for help in academic cheating, from 88.8% to 26.9% depending on a cheating behaviour. Students would most often ask a family member or friend for help in academic cheating. The same “helpers” were identified for non-academic related behaviour – asking for personal material favours. More respondents, however, would include three or four persons for asking help in academic cheating than for routine material favours. Score on material favours survey was the strongest positive predictor of readiness for asking help in academic cheating (stepwise MLR model; beta = 0.308, P < 0.0001) followed by extrinsic motivation (compensation) and male gender, whereas intrinsic motivation, year of study and grade point average were weak negative predictors.

**Conclusions:**

Our study indicates that medical students are willing to engage more than one person in either close or distant relationships in academic cheating. In order to develop effective preventive measures to deter cheating at medical academic institutions, factors surrounding students’ preference towards academic cheating rather than routine favours should be further investigated.

## Background

There is growing evidence that academic dishonesty is widespread in medical and health care schools worldwide [1-5]. This has a detrimental effect on medical practice because students who cheat during medical school follow the same behavioural pattern later on in their work with patients [6-9]. Finally, social predictors such as socioeconomic environment and educational system have been shown to have an important influence, most notably in post-communist countries in Europe [10]. We and other research groups in Croatia have demonstrated that academic dishonesty was acceptable behaviour among medical students [3,11-13] and that they came to medical schools ready to cheat [14], indicating that different familial and cultural values acquired long before medical school have had an impact on student behaviour.

Academic cheating most often is a collaborative practice as opposed to an isolated act by an individual. However, there are few studies that examined factors that looked at the persons who are actually involved or would be potentially engaged in collaborative academic cheating. Surveys that investigated attitudes towards engaging others in cheating use general terms such as a “colleague”, “peer”, “another student”, “someone else”, and sometimes a “friend” [4,5,15,16], although it can be hypothesized that the view of those involved in collaborative cheating would be different for less conspicuous than for more serious academic transgressions. For example, it could be considered risky to collaborate with strangers in serious cheating behaviour, which would be asked only from individuals in a close relationship. The aim of the present study was to address the following research questions: 1) what is the readiness of medical students to engage others in academic dishonest behaviours; 2) who would students ask for help in academic cheating; and 3) is it more acceptable to ask more individuals for help with routine material favours than it is to seek help with academic cheating? The willingness for including others in academic cheating was also correlated with individual factors described as potential predictors of student cheating [10]: gender, year of study, academic success and motivation for study/work.

## Methods

### Participants

We performed a cross-sectional study at the University of Zagreb School of Medicine in Croatia in 2008. The study population included medical students attending the 1st, 3rd and 6th (last) year of study. They were asked to voluntarily complete an anonymous, self-administered questionnaire about their propensity for cheating and motivation. The questionnaire was distributed to a convenience sample of all students present at lectures or seminars when the highest student presence was expected. The same approach and introductory lines by investigators were used for all groups tested. The study was approved by the Ethics Committee of the University of Zagreb School of Medicine.

### Questionnaire

The questionnaire consisted of three parts: general data, questionnaire about students’ propensity to ask for favours and the Work Preference Inventory (WPI) [17].

General questions included age, gender, grade point average (GPA) at the final year of secondary school for the 1st year students or GPA at the previous year for the 3rd and 6th year students.

The questionnaire tested students’ willingness to ask for unethical favours in academic situations and their readiness to ask for material personal favours in everyday situations from 4 different types of individuals: 1) family member or close relative; 2) best friend; 3) colleague; and 4) stranger.

Academic favours were chosen from each of the four specific clusters from the hierarchical cluster analysis of 11 self-reported cheating behaviours at the same school [11]: ask another person to sign the lecture attendance sheet instead of the respondent (A1); ask another person to let the respondent copy answers during a written exam (A2); ask another person to send answers to the respondent during a test, using a cell phone (A3); and ask another person to use private connections with the examiner to arrange a passing exam grade with the examiner (A4). The favours were listed from the least to the most serious transgression.

Personal material favours presented to the respondents were: lending the respondent €50 for three days (P1) and lending a car for a day (P2).

A pilot test was conducted for the part of the questionnaire that related to favours was piloted with 4 educators and students from the University of Zagreb School of Medicine in order to improve the clarity, consistency and face validity of the questionnaire.

The third part of the questionnaire was the Work Preference Inventory (WPI) for assessing individual differences in intrinsic and extrinsic motivational orientations of college students [17]. The WPI consists of 30 statements in which a respondent is asked to choose a single statement that expresses to what extent that statement described her/him: 1. Never or almost never; 2. Sometimes; 3. Often; and 4. Always or almost always. The questionnaire was scored on two primary scales (intrinsic and extrinsic motivational orientation, each with 15 statements), each subdivided into 2 subscales: *outward* and *compensation* for extrinsic motivation and *enjoyment* and *challenge* for intrinsic motivation (10 and 5 statements, respectively). The score range for each statement was from 1 (completely disagree) to 4 (completely agree).

### Data analysis

The frequencies of positive answers indicating students’ willingness to ask others for help in academic cheating and their readiness to ask the same persons for personal favours were analyzed separately for each favour. Average scores per item were generated by summing positive answers for academic or personal favours and dividing them by the number of items in the scale, so that the overall scores had the possible range from 0 to 1 indicating the average per-item fraction of positive answers. Differences in the mean per-item score of academic favour requests between genders and study years were tested by Student’s *t*-test and ANOVA, respectively, whereas nonparametric counterparts, Mann-Whitney and Kruskal-Wallis tests, were used to estimate gender and year of study median differences in per-item score for personal favours. The binomial test was used to compare the expected with observed frequency of positive answers.

Motivation scores were expressed as mean (± standard deviation, SD) per-item scores on each of the two scales (intrinsic and extrinsic) and four subscales (enjoyment, challenge, outward, compensation). Summative scores on the scales were divided by the number of items in each scale, so that the overall scores had a possible range from 1 to 4, making comparisons between the scales possible.^17^ The mean scores for an item in the motivation scale were tested between genders using Student’s *t*-test, whereas differences among the years of study were tested with ANOVA and subsequent Bonferroni post-hoc analysis. Cronbach’s alpha was used to estimate internal consistency of all scales and subscales used in the study.

Stepwise multiple linear regression models of the summed score favour requests with gender, year of study, repetition of the year, grade point average and motivational subscores as independent variables, were used to evaluate potential factors influencing propensity for engaging others in cheating. In the model of personal favours, score data were first logarithmically transformed to correct for non-normality of error distribution.

Statistical analyses were performed using MedCalc, version 12.5 (MedCalc Software, Ostend, Belgium). The level of significance was set at P < 0.05.

## Results

### Respondents

The total number of respondents was 591, with 245 (41.6%) 1st year students, 196 (33.1%) 3rd year students, and 150 (25.3%) 6th sixth year students. The response rates were 83.1%, 71.3% and 63.3%, respectively. Most of the respondents were women, reflecting the typical gender structure of the school [11], and had high grade point averages (Table 1).

**Table 1:** **Characteristics of the study population**

**Characteristics**	**No. (%)**
Gender:	
Women	387 (65.5)
Men	196 (33.2)
Missing data	8 (1.3)
Study year:	
1st	
Women	169 (69.0)
Men	76 (31.0)
3rd	
Women	120 (61.2)
Men	73 (37.2)
Missing data	3 (1.6)
6th	
Women	98 (65.3)
Men	47 (31.3)
Missing data	5 (3.4)
Grade point average:	
1st year*	4.8 ± 0.2
3rd year	4.0 ± 0.5
6th year	4.1 ± 0.4

*Grade point average for the first year of study was the GPA of the students at the high-school, which is required for the entrance to the University.

### Asking for academic and personal favours

We first assessed the internal consistency of the academic cheating survey. Overall Cronbach’s α for 16 items in the academic cheating survey (4 cheating situations × 4 types of persons to be engaged in the cheating act) was 0.850, 95% confidence interval [CI] 0.832 – 0.868, demonstrating a high level of internal consistency. For the personal favours survey, the overall Cronbach’s α was 0.723, 95% CI 0.687 – 0.756 due to a smaller number of items (n = 8) and also indicated a high level of consistency.

Additionally, in both surveys all items had acceptable item-total correlations of r_pb_ ≥ 0.317 and no item had a Chronbach’s α-if-item-deleted value greater than the overall α.

Proportions expected by chance were used to analyze individual cheating situations. In case of no social constraints in a particular situation, we could presume an equal probability of asking any of the offered individuals (family, friend, colleague, and stranger). As there were 16 possible combinations of individuals to ask, the probability per random combination would be 6.25%. Observed and expected proportions of responders with intention to engage a particular number of persons in a cheating behaviour are presented in Table 2. All observed proportions differed significantly from those expected by chance alone. In particular, for all academic cheating situations (A1–A4), the frequency of the choice not to include anyone was significantly higher than expected, thus indicating the awareness of students that the behaviour was not permitted and/or acceptable. Likewise, the choice of respondents not to ask anyone for a personal material favour was also more frequent than expected suggesting responders’ restraint in asking for material favours. Complementary results were observed with responders intending to engage only a single person in a particular behaviour, either the academic or non-academic, as observed proportions were significantly lower than expected, again indicating a restraint in asking. Academic cheating, however, was shown as a more cooperative process since significantly more responders than expected would include either all four (questions A1–A3) or three persons (A1) in dishonest behaviour. Contrarily, when asking for personal material favours respondents would predominantly engage two persons – exclusively a friend and a family member.

**Table 2:** **Observed and expected proportions of responders with intention to engage different number of persons in a cheating behaviour**

**Question**	Observed proportions, No. (%)* for engaging:				
	**None**	**One person**	**Two persons**	**Three persons**	**Four persons**
**Proportion expected by chance**	6.3%	25.0%	37.5%	25.0%	6.3%
**Academic cheating:**					
A1. Sign lecture attendance sheet	64 (11.2%)	66 (11.6%)	169 (29.6%)	171 (30.0%)	100 (17.5%)
A2. Let copy answers during test exam	93 (16.1%)	66 (11.4%)	149 (25.7%)	106 (18.3%)	165 (28.5%)
A3 Send answers by cell phone during test exam	288 (50.0%)	26 (4.5%)	151 (26.2%)	56 (9.7%)	55 (9.5%)
A4. Use personal connection to pass exam	422 (73.1%)	42 (7.3%)	81 (14.0%)	16 (2.8%)	16 (2.8%)
**Personal favours:**					
P1. Lend car for a day	98 (17.0%)	113 (19.6%)	336 (58.1%)	11 (1.9%)	20 (3.5%)
P2. Lend € for three days	111 (19.3%)	112 (19.4%)	313 (54.3%)	17 (3.0%)	23 (4.0%)

*Observed proportions were calculated in relation to the total number of respondents who answered the questions. All proportions significantly differed from values expected by chance, *P* ≤ 0.007.

**Figure 1:**
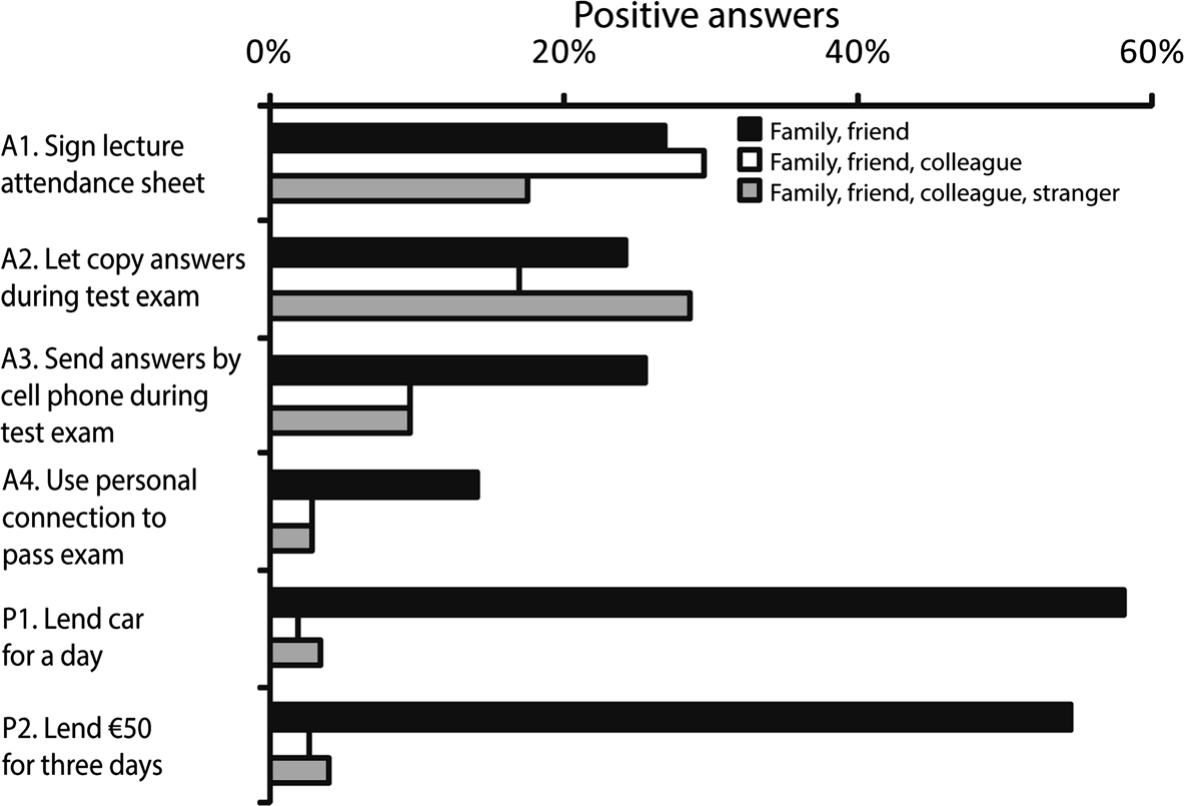
**Frequency of medical students’ (n = 592) willingness to engage others (family, friend, colleague or stranger) in unethical academic behaviours (A1–A4) or routine material favours (P1–P2).**

Regarding a particular relationship with engaged persons, responders asking only a single person would engage a friend more often than expected for questions A1 (10.5% out of 11.6% single-person choices) and A2 (8.2%) (binomial test, *P* < 0.001 for all), whereas the engagement of other persons was rare. For all academic transgressions including two individuals, the choices which were overall underrepresented, “family and friend” was chosen significantly more often than expected for a random combination (26.8%, 24.2%, 25.5% and 14%, binomial test, *P* < 0.001 for all, Figure 1). Likewise the observed frequencies for the choices of three individuals for the involvement in academic cheating were significantly higher than the expected only for the choice of “family, friend, or colleague”. This was observed for all academic transgressions except the most serious one of asking someone to arrange passing of the exam with the professor which was overall rare (29.5%, 16.9% and 9.4% for A1–A3, respectively, *P* < 0.001, Figure 1). In the same academic transgressions the frequency of extending the circle of individuals involved to “family, friend, colleague, and stranger” was also significantly higher than expected (17.5%, 28.5% and 9.5% for A1–A3, respectively, *P* < 0.001, Figure 1).

For personal material favours, the respondents would predominantly ask one or two persons. For a choice of single person it was predominantly “family” (16.7% and 13.7% for P1 and P2, respectively), whereas for a choice of two persons it was exclusively family or friend (Table 2, Figure 1). Only a single respondent chose somebody else for lending a car.

The proportion of students who would ask for unethical academic favours depended on the seriousness of the offence: 88.8% of them were ready to ask at least one person (either in close or distant relationship) to sign a lecture attendance sheet in their place, 83.9% would ask someone to copy test answers during an exam, 50.0% would ask for test answers sent by a cell phone, and 26.9% would use someone’s connection with the teacher to pass an exam. The percentage of students who would ask someone for a non-academic favour (83.0% for lending a car and 81.7% for lending money) was similar to that of asking for the least offensive academic transgressions.

### Who are the cheaters?

Male respondents were significantly more prone to ask for either an academic transgression or a material personal favour than female respondents only in the first year of study (Table 3). Differences across study years were found only for female students in the 3rd year of the study, as these students would significantly more often engage in academic cheating collaboration than the 1st year female students: ANOVA for differences across study years: F_2,365_ = 4.27, *P* = 0.015; post-hoc for 1st vs. 3rd year: *P* = 0.012 (Table 3).

**Table 3:** Average per-item scores* for unethical requests (mean ± standard deviation) with respect to study years, type of requests, and gender of medical students

**Favours asked**	**Study year**	**Men**	**Women**	***P*** **-value (gender comparison)** ^**†**^
**Academic cheating**	1st	0.42 ± 0.27	0.35 ± 0.23	0.041
	3rd	0.45 ± 0.24	0.43 ± 0.21^‡^	0.574
	6th	0.37 ± 0.21	0.39 ± 0.21	0.548
**Personal favours**	1st	0.45 ± 0.24	0.37 ± 0.17	0.035
	3rd	0.40 ± 0.26	0.38 ± 0.22	0.751
	6th	0.42 ± 0.16	0.34 ± 0.17	0.002

*Scores were expressed as summative scores on the scales divided by the number of items in each scale, resulting in the possible range from 0 to 1.

^†^Student *t*-test.

^‡^Statistically significant difference vs. 1st year (*P* = 0.012, ANOVA, posthoc).

### Respondents’ motivation for work/study

The total sample of medical students had higher intrinsic than extrinsic motivation (mean item score on 1–4 summary scale 3.07 ± 0.36 vs. 2.67 ± 0.36, respectively). Female students had higher intrinsic motivation than male students (3.09 ± 0.34 vs. 3.02 ± 0.39, *P* = 0.0048, *t*-test), due to higher scores on the Enjoyment subscale (3.34 ± 0.35 vs. 3.20 ± 0.41, *P* < 0.001). There were no differences in their extrinsic motivation, but male students had higher scores on the Compensation subscale (2.72 ± 0.52 vs. 2.56 ± 0.49, *P* < 0.001). Across study years, intrinsic motivation significantly decreased in the 3rd as opposed to the 1st year of study (2.99 ± 0.41 vs. 3.11 ± 0.31, ANOVA post-hoc *P* = 0.002), mostly because of a significant decrease in the Enjoyment subscale, but increased again at the end of medical school. The overall extrinsic motivation did not change over the years, although there was a significant decrease in the Outward subscale in higher years relative the 1st year of study (2.77 ± 0.39 vs. 2.66 ± 0.41 vs. 2.63 ± 0.36, respectively, ANOVA *P* = 0.001, post hoc tests for comparison with 1st year *P* from 0.003 to 0.009).

### Predictors of willingness to ask favours

We found that the personal favours score was the strongest and highly significant predictor of willingness in asking others for help in academic cheating, with students having higher scores on this construct more likely engaging in dishonest behaviour (Table 4). In addition, students who had higher GPAs, were enrolled in a higher year of study and had higher Intrinsic motivation (Enjoyment subscale) also had a lower willingness to engage others in academic cheating, while higher extrinsic motivation on the Compensation subscale was a positive predictor for such behaviour (Table 4). Whereas the weighted combination of all predictor variables explained approximately 18% of the variance in the willingness for involving others in academic cheating, the personal favours score alone explained up to 9.4% of the variance. In contrast, male gender and year of study were weak independent predictors (positive and negative, respectively) of readiness for asking for personal favours, in a model that described only 3.6% of the variance (Table 4).

**Table 4:** **Predictors of respondents’ (n = 592) readiness for ask for help in academic cheating or for a personal favour, ordered by the predictor strength from strongest to weakest**

**Request**	**Significant predictor**	**Beta**	***P***	**Interpretation**
**Academic cheating** adjusted R^2^ = 18.0%	Score on personal favours scale	0.308	3.7 x 10^−8^	Higher readiness, more requests
	Intrinsic motivation	−0.197	4.1 x 10^−4^	Higher enjoyment, fewer requests
	Extrinsic motivation – Compensation	0.191	0.001	Higher compensation, more requests
	Grade point average (GPA)	−0.113	0.039	Higher GPA, fewer requests
	Gender	0.119	0.033	Male gender, more requests
	Year of study	−0.113	0.041	Higher year of study, fewer requests
**Personal favours** adjusted R^2^ = 3.6%	Gender	0.164	0.008	Male gender, more requests
	Year of study	−0.123	0.046	Higher year of study, fewer requests

## Discussion

Our study demonstrated that medical students were willing to involve others in academic cheating. Personal material favours, such as asking to borrow some money or a car, were predominantly reserved for family and friends, similar to the most serious offence in academic cheating – asking someone to use a personal connection with the examiner to arrange a passing exam grade for the respondent. There was a clear gradation of academic misbehaviours for which students would ask persons outside of the narrow circle of family and friends. Students had no problems in engaging strangers in academic cheating, especially for favours they considered less serious, such as signing a lecture sheet or copying answers during a test. There were no major differences across study years and genders.

The limitations of our study include the self-reported nature of the data collected on cheating and the fact that we asked them about their intentions and not actual conduct, as it is not clear whether asking about future intentions is a more sensitive indicator of cheating than directly requesting a report on past cheating [11]. Also, the results of the survey may have been affected by a potential overlap in the meaning (a “friend” who could also be a “colleague”). In the Croatian language, in which the survey was performed, the difference is clear and respondents had the ability to select the “friend” response to describe their friendly relationship with a fellow medical student. The responders’ choices for personal favours (exclusively family or friend) indicated that these categories were not confused. Furthermore, the study was performed in a single medical school, limiting the generalizability of our findings. However, the response rate across all study years in this study was high, and the responses obtained about their willingness to ask others for help in cheating were similar to reported prevalence of admitted cheating behaviour in the same and other medical schools in Croatia over the last decade [3,11]. For example, the prevalence of admitted academic cheating of medical students for the four academic misbehaviours that were included in this study were 89.1% for signing for someone else on the attendance sheet, 52.2% for copying answers during a test and 1.6% for using private connections to pass an exam [11]. The consistency of data over time confirms that the phenomenon of high academic cheating in the observed setting is real and well-established, confirming the relevance of the results of the current study on students’ willingness to ask for help in cheating to a real-world situation at similar academic institutions.

The major predictor for engaging others in academic cheating was the score on readiness to ask someone for a personal favour, which explained a half of the total variance in the regression model that also included intrinsic and extrinsic motivation, gender, grade point average and year of study. On the other hand, the only positive predictor for personal favours was male gender.

Taken together, these results indicate that academic cheating is perceived by medical students as an activity mostly outside of one’s personal accountability. In an environment that is very permissive to academic cheating and cheating in general [3,10-14], students’ choice of persons who they would be ready to involve in cheating demonstrates their awareness of the acceptability of the behaviour. For smaller academic transgressions they would not mind asking a favour from anyone available, including strangers. As the seriousness of cheating increases, they were more selective in their choice of individuals, being aware of the transgression and the consequences for the other person. It is important to emphasize that our study did not address academic collusion, which is considered as a cheating behaviour when students engage in collaboration and is defined as an “unacceptable level of shared work in the final assignment” [18]. All of the behaviours used in our study survey were clear transgressions of individual behaviours and specified in the curricular norms of the institution [11].

Social and educational system predictors of academic cheating are particularly strong in post-communist countries in Europe, not only in medicine but also in other disciplines, such as economy and business, leading to drastic differences in cheating: the prevalence of academic cheating in these disciplines was 88% in Eastern European countries compared with 5% in Scandinavian countries [10]. A recent study from Iran, using a randomized response technique to control for responses to sensitive issues, demonstrated that 93% of medical students impersonated an absent student during a mandatory class [15]. We showed in our previous report from 2004 that 94% of the students from the same medical school as in the current study admitted to cheating at least once during their studies [11]. Comparing the data from this study to those from our 2004 study, students’ attitudes towards cheating have become even more lax. Academic misbehaviour offered to respondents were chosen from 4 very distinct clusters of academic transgressions, based on reported behaviour, approval of such behaviour, perception about its frequency and willingness to report cheating. In the current study, the prevalence of possible engagement of others in the least serious offenses (signing an attendance sheet and copying during a written exam) was similar (88.8% and 83.9%), whereas these differences were higher for their respective clusters in the previous study (87% and 56%, respectively for the perception that such behaviours are common). A multi-campus study of medical schools in Croatia showed that not only did almost all of the freshmen admit to cheating, but most of them enrolled with cheating experience from high schools [14]. Croatian medical students also exerted pressures on and engaged others in asking their teachers for undeserved academic benefits, as 45% of the medical teachers reported experiences of pressures intended to ensure that the student obtains a passing or a better grade on an exam [19].

While previous studies suggested that men generally self-reported more cheating than women [8], this study and other studies from Croatia [3,11] did not find major gender differences. In our study, female students had less willingness in engaging others in academic cheating only in the 1st year of study and asked fewer personal favours. However, gender was a weak predictive factor in multiple linear regression model, with male students being more willing to ask either for a personal favour or help in cheating. The lack of major gender differences can be explained by long-standing female predominance at medical schools in Croatia [3,11].

On the other hand, the only change that occurred during the course of the medical curriculum was that female students showed greater willingness for engaging others in academic cheating in the 3rd year then when they began medical education. This may be due to the fact that female students often adopt the male pattern of behaviour when engaged in a traditionally male predominant profession [20], particularly in the setting of clinical teaching at university hospitals, which starts in the 3rd year and is dominated by men, particularly in leadership positions [21]. The 3rd year, where we observed the highest propensity for engaging others in cheating, was also characterized by a significant decrease in intrinsic motivation of the students in our study. This finding may be related to the pressures to be successful while facing the difficulties of medical education and the strong hierarchy of clinical work, which may have affected their behaviour. Our previous study of moral reasoning in medical students showed that moral reasoning deteriorated in the third year of the curriculum, when students enter the clinical rotations [22].

The finding that the study year, gender, GPA and motivation were weak predictors of the willingness to engage others in cheating in the multiple linear regression model indicates that collaboration in cheating at medical schools is predominantly influenced by the contextual and social settings [8,10]. This means that interventions aiming at improving individual integrity of the students, such as those related to their academic success or fostering of intrinsic motivation for study and work, cannot be effective without structural changes in the institutions, educational system and society in general. Students are also aware of this, as recently reported by Henning et al. [5], who performed a qualitative study of reasons for cheating and suggestions for strategies to decrease cheating among medical and pharmacy students. The great majority of students (91%) expected and were willing to respond to cheating control by external bodies, from parents and teachers to institutions as well as agencies and governments [5].

## Conclusion

Our study demonstrated that medical students are willing to ask others for help in academic cheating. Academic cheating is a part of a wider human network that supports corruption in societies [23], and translates into corruption in the health sector which can then lead to a perception of lower quality health care [24]. In the situation of increased migrations of health workers within Europe and globally [25], the social aspect of willingness to engage others in academic cheating and the consequences of such behaviour are not only the problems of individual schools or countries but need to be addressed at the societal level.
